# Colorimetric Assay Reports on Acyl Carrier Protein Interactions

**DOI:** 10.1038/s41598-019-51554-6

**Published:** 2019-10-30

**Authors:** Kofi K. Acheampong, Bashkim Kokona, Gabriel A. Braun, Danielle R. Jacobsen, Karl A. Johnson, Louise K. Charkoudian

**Affiliations:** 1 Department of Chemistry, Haverford College, Haverford, PA 19041-1391 USA; 2 Department of Biology, Haverford College, Haverford, PA 19041-1391 USA

**Keywords:** Carrier proteins, Enzymes, Multienzyme complexes

## Abstract

The ability to produce new molecules of potential pharmaceutical relevance via combinatorial biosynthesis hinges on improving our understanding of acyl-carrier protein (ACP)-protein interactions. However, the weak and transient nature of these interactions makes them difficult to study using traditional spectroscopic approaches. Herein we report that converting the terminal thiol of the *E*. *coli* ACP 4′-phosphopantetheine arm into a mixed disulfide with 2-nitro-5-thiobenzoate ion (TNB^−^) activates this site to form a selective covalent cross-link with the active site cysteine of a cognate ketoacyl synthase (KS). The concomitant release of TNB^2−^, which absorbs at 412 nm, provides a visual and quantitative measure of mechanistically relevant ACP-KS interactions. The colorimetric assay can propel the engineering of biosynthetic routes to novel chemical diversity by providing a high-throughput screen for functional hybrid ACP-KS partnerships as well as the discovery of novel antimicrobial agents by enabling the rapid identification of small molecule inhibitors of ACP-KS interactions.

## Introduction

Microorganisms house modular enzyme-based factories that generate molecules of astounding structural diversity and exquisitely tuned biological activities. Acyl carrier proteins (ACPs) play a central role within these assembly lines by covalently tethering building blocks and intermediates as thioesters linked to a conserved 18 Å 4′-phosphopantethiene (Ppant) arm and faithfully shuttling these molecular components to appropriate catalytic partners to perform the programmed chemical reactions. Understanding the molecular recognition features of ACPs that guide their interactions with partner enzymes is important for at least two reasons. First, the ability to gain access to expanded chemical diversity via combinatorial biosynthesis hinges on building hybrid synthases in which the ACP is capable of interacting with non-cognate partners. Second, because ACPs are involved in primary metabolism pathways, such as the *E*. *coli* fatty acid synthase (FAS) pathway, understanding the molecular underpinnings of ACP-protein interactions can lead to the identification of antibiotics by rational design or by screening of combinatorial libraries that inhibit these essential protein interactions. Despite the clear importance of understanding ACP-protein interactions, progress has been historically stymied by the lack of spectroscopic approaches amenable to capturing the rapid and weak interactions of the ACP.

**Figure 1:**
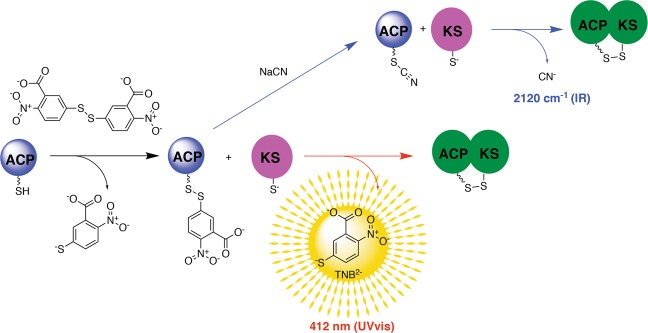
The discovery of a colorimetric assay that reports on ACP-KS interactions. While investigating ACP-KS interactions using a previously reported vibrational spectroscopic mechanistic cross-linking approach (blue arrows), an unexpected color change was observed upon mixing the ACP substrate with the KS partner. Upon investigation, it was revealed that the cyanylation reaction had not gone to completion, and thus instead of the ACP-thiocyanate being mixed with KS, the ACP-TNB^−^ complex was inadvertently added. This led to the realization that the facile activation of ACP to ACP-TNB^−^ enables the colorimetric reporting of mechanistically relevant ACP-KS interactions (red arrows).

One way to overcome obstacles associated with the transient interactions of ACPs is to utilize chemical probes to trap ACP-protein interactions^1^. Trapping the ACP-ketoacyl synthase (KS) interaction is of particular interest, as this partnership is essential in facilitating the chain elongation and chain transfer reactions that build the carbon skeleton of any polyketide or fatty acid product. Successful processing by the KS relies on both proper substrate recognition and productive ACP-KS protein interactions^2,3^. Pioneering work by Burkart and co-workers revealed that the incorporation of an electrophile on the terminus of the ACP Ppant arm can serve as a warhead that traps the mechanistically relevant ACP-KS complex through a substitution reaction with the nucleophilic thiolate in the active site of the KS^4^. We later reported that converting the terminal thiol of the Ppant arm to a thiocyanate provides a site-specific vibrational spectroscopic probe for ACPs that reports on the Ppant arm conformational dynamics of the ACP itself^5^. In the presence of a compatible KS, the thiocyanate-labeled ACP is activated to form a mechanistically relevant cross-link through disulfide bond formation between the Ppant arm thiol and partner KS thiolate^6^. The concomitant release of CN^−^ enables the monitoring of ACP-KS interactions via infrared (IR) spectroscopy (Fig. 1). These methodological advancements enable structural biology and protein engineering efforts to interrogate the molecular underpinnings of ACP-KS interactions.

In the context of a Course-based Undergraduate Research Experience (CURE), we embarked on a 14-week project in which undergraduate students proposed to utilize the thiocyanate cross-linking approach to investigate the molecular basis of the *E*. *coli* FAS ACP, AcpP, interaction with a cognate KS, FabF. Each student designed, constructed, expressed and purified a distinct mutant ACP to convert to the thiocyanate version and assess via mechanistic cross-linking. In addition, students expressed and purified wild type AcpP as a positive control along with the ACP from the actinorhodin type II PKS (ACT ACP) as a negative control since this protein does not bind to FabF *in vitro*^6–8^. One student observed that their reaction turned yellow upon mixing their thiocyanate-labeled AcpP mutant with the KS. As a class, we decided to determine the origins of this unexpected result and first analyzed more closely the starting materials for the reaction. Inspection of the thiocyanate-labeled AcpP by mass spectrometry revealed that the thiol of the Ppant arm of the AcpP mutant was not fully converted to the thiocyanate, but rather was in the 2-nitro-5-thiobenzoate (TNB^−^) intermediate state (Fig. 1). We recognized the potential power of this unexpected result: the incubation of the ACP-TNB^−^ adduct with KS led to the mechanistically relevant cross-linking between the two enzymes and the concomitant release of TNB^2−^, which provided a colorimetric readout (Fig. 1). Herein we report the details of this discovery and showcase the application of this methodology to combinatorial biosynthesis and bacterial inhibitor design. The approach described in this paper offers real advantages over previous work^1,4,6–9^ in that it adds a colorimetric readout that can be used to monitor the cross-linking reaction and screen for small molecule inhibitors, while eliminating additional steps involving expensive and toxic reagents.

## Results

### Purification of holo-ACPs and FabF

AcpP, ACT ACP and FabF were expressed as His_6_-tagged constructs in BAP1^10^ or BL21-DE3 competent cells. We originally intended to convert the terminal thiol of the ACP Ppant arm to the thiocyanate for subsequent mechanistic cross-linking and monitoring via infrared spectroscopy, and thus it was essential to obtain the ACPs of interest in the *holo*-form (in which the Ppant arm is installed). Despite the capability of BAP1 cells to co-express the Sfp phosphopantethienyl transferase that catalyzes the transfer of a 4′-Ppant moiety from CoA to the conserved serine residue at the *N*-terminus of helix II of ACP to afford *holo*-ACP, we obtained mixtures of *apo*- and *holo*- ACPs from protein expression in this cell line. The observed mixtures could be due to inefficiencies of the BAP1 cell line or the presence of AcpH, a phosphodiesterase that cleaves the Ppant arm in *E*. *coli*^11,12^. The presence of AcpS, a native phosphopantetheinyl transferase in *E*. *coli*, likely explains why some *holo*-ACP also was obtained from the BL21-DE3 cell line. To ensure that ACPs were in a majority *holo*-form, Ni-bound ACPs were chemoenzymatically treated *in vitro* with SfpR4-4^13^ in the presence of coenzyme A before the elution step during purification. SfpR4-4 is a mutant of the *Bacillus subtilis* Sfp discovered by high-throughput phage selection that displays a 300-fold increase in catalytic efficiency and broader substrate specificity than the wild-type Sfp^13^. The successful conversion to 100% *holo*-ACP was confirmed by liquid chromatography mass spectrometry (LCMS) and protein purity evaluated by SDS PAGE (Figs S1–S3). *Holo*-AcpP was observed to migrate to ~20 kDa on SDS PAGE despite its molecular weight of ~10 kDa (Fig. S3). This is consistent with previous observations and is likely due to the unusual charge distribution of the protein^14^. A second higher molecular weight band at ~40 kDa observed on non-reducing gels represents a dimer that forms via the disulfide bond between the Ppant arm thiols of monomeric ACPs^15^. *Holo*-ACT ACP migrated to the expected molecular weight of ~10 kDa on SDS PAGE gel and also exhibited dimer formation under non-reducing conditions (Fig. S3). The migration of FabF on SDS PAGE gels was consistent with the monomeric mass (~45 kDa), although it may exist as a dimer under native conditions (see below).

### Investigation of AcpP and ACT ACP oligomeric state and capacity of these proteins to bind to the KS FabF

SV-AUC measures the sedimentation rate of molecules or proteins in solution and can provide information about molecular shapes, oligomeric state and binding affinity. We observed that the sedimentation profiles of AcpP and ACT ACP fitted to a model-free continuous c(s) distribution. AcpP (12.3 kDa) sediments with a broad (9.97–12.8 kDa) major peak centered at s(20,w) = 1.7 S, whereas ACT ACP (11.4 kDa) sediments slightly differently with a major peak at s(20,w) = 1.5 S. This observation suggests that AcpP sediments at an apparently “larger” molecular radius, which is consistent with how the protein behaves in SDS-PAGE and in solution (Fig. S4)^15^. Nonetheless, it appears that both ACPs exist primarily in a monomeric state under reducing conditions. It appears that AcpP forms a tetramer (Fig. S4), but whether this self-association is physiologically relevant remains to be investigated.

**Figure 2:**
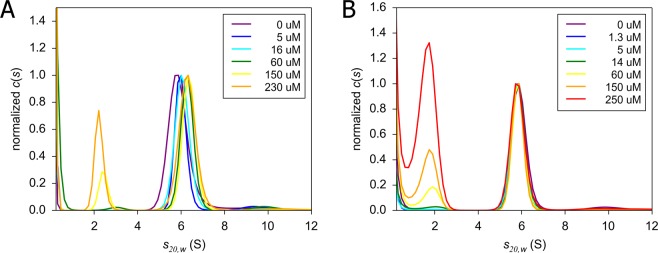
*Holo-*AcpP interacts with *E*. *coli* KS FabF, whereas *holo*-ACT ACP does not. The SV-AUC titration profile of constant 16 μM FabF with increasing amounts of AcpP shows an initial increase in s-value and then remains constant (A). Given the transient nature of interactions, the AcpP-FabF complex is likely in equillibrium with free FabF. At high concentrations, excess free AcpP is observed at lower s-values. In contrast to AcpP, titration of FabF with *holo*-ACT ACP shows no shift in the peak position for the FabF (**B**).

Next, we used SV-AUC to study ACP-FabF interactions by looking for significant changes in the single boundary of the sedimentation velocity profile of FabF at varying concentrations of ACP. Prior to measuring the binding affinity, we first determined that FabF sediments mostly as a dimer in solution (Fig. S4), which is consistent with previous crystallography studies^16^. We observed that the FabF sedimentation boundary changes upon addition of AcpP but remains unchanged when ACT ACP is added (Fig. 2). These data suggest that AcpP complexes with FabF whereas ACT ACP does not, which is consistent with previous *in vitro* cross-linking studies^6–8^.

To measure the binding strength between FabF and AcpP, we applied analysis of weighted-average (signal average) sedimentation coefficient (s_w_) as a function of AcpP concentration. Partial saturation of the reaction boundary was achieved by titrating 16 μM FabF with up to 230 μM AcpP (Fig. S5). An EPT s(w) fast isotherm was constructed by defining the reaction boundary between 4 and 8 S, and a second isotherm was constructed by integrating the c(s) distribution between 1 and 8 (Fig. S5). The k_D_ value of 6.4 ± 2.0 μM was obtained when both isotherms were fit globally to an A + B ↔ AB model by fixing free FabF at 5.9 S, AcpP at 2.0 S and fitting to a convergent dimer of heterodimers s-value of 6.6 S (Fig. S5). The calculated k_D_ is in close agreement with the k_D_ of 4.1 ± 1.8 μM previously reported using isothermal calorimetry^9^, validating SV-AUC as a method to quantify ACP-FabF interactions in solution.

### Conversion of Holo-ACPs to ACP-TNB^−^

After using SV-AUC to establish that FabF binds to *holo*-AcpP but not to *holo*-ACT ACP, we sought to characterize these same interactions using the coupled mechanistic cross-linking reaction/vibrational spectroscopic approach developed in our lab^6^. To install the thiocyanate probe on the terminal thiol of the Ppant arm of *holo*-AcpP and *holo*-ACT ACP, we first needed to convert the *holo*-ACPs to their corresponding ACP-TNB^−^ adducts. *Holo*-ACPs were treated with excess 5,5′-dithiobis-(2-nitrobenzoic acid) (DTNB), commonly known as Ellman’s reagent^17^, to install the TNB^−^ probe on the terminal thiol end of the 4′-Ppant arm of *holo*-ACPs. To avoid a second TNB^−^ probe adding to a cysteine residue near the *N*-terminus of ACT ACP, the C17S ACT ACP construct was utilized. A bright yellow color signifying the release of half of DTNB (TNB^2−^ ion) into solution was observed when the *holo*-ACPs reacted with DTNB. The ACP-TNB^−^ products were purified via size exclusion chromatography (SEC) with a PD10 column and subsequently characterized by LCMS (Figs S1 and S2). Comparison of the *holo*-ACP to ACP-TNB^−^ circular dichroism (CD) spectra revealed that the installation of the TNB^2−^ molecule to the Ppant arm did not perturb the overall helical structure of the protein (Fig. S6).

### AcpP-TNB^−^ forms a mechanistically relevant cross-link with the active site cysteine of FabF

In the course of generating and using the AcpP-SCN product for mechanistic crosslinking with FabF^6^, it was noted that a reaction evolved a bright yellow color upon mixing, reminiscent of the color change when TNB^2−^ is released. This observation led us to hypothesize that the unexpected color change was driven by the KS active site thiolate acting as a nucleophile and attacking the thiol-bound TNB^−^ adduct, as outlined in Fig. 1. Upon analysis of the starting material by LCMS, we confirmed that in this case the reaction of the ACP-TNB^−^ with sodium cyanide had not gone to completion and thus the AcpP-TNB^−^ adduct had been mixed with FabF. Recognizing the potential fortuitous discovery of a facile method to obtain a mechanistically relevant KS-ACP complex, we further investigated this unexpected result.

**Figure 3:**
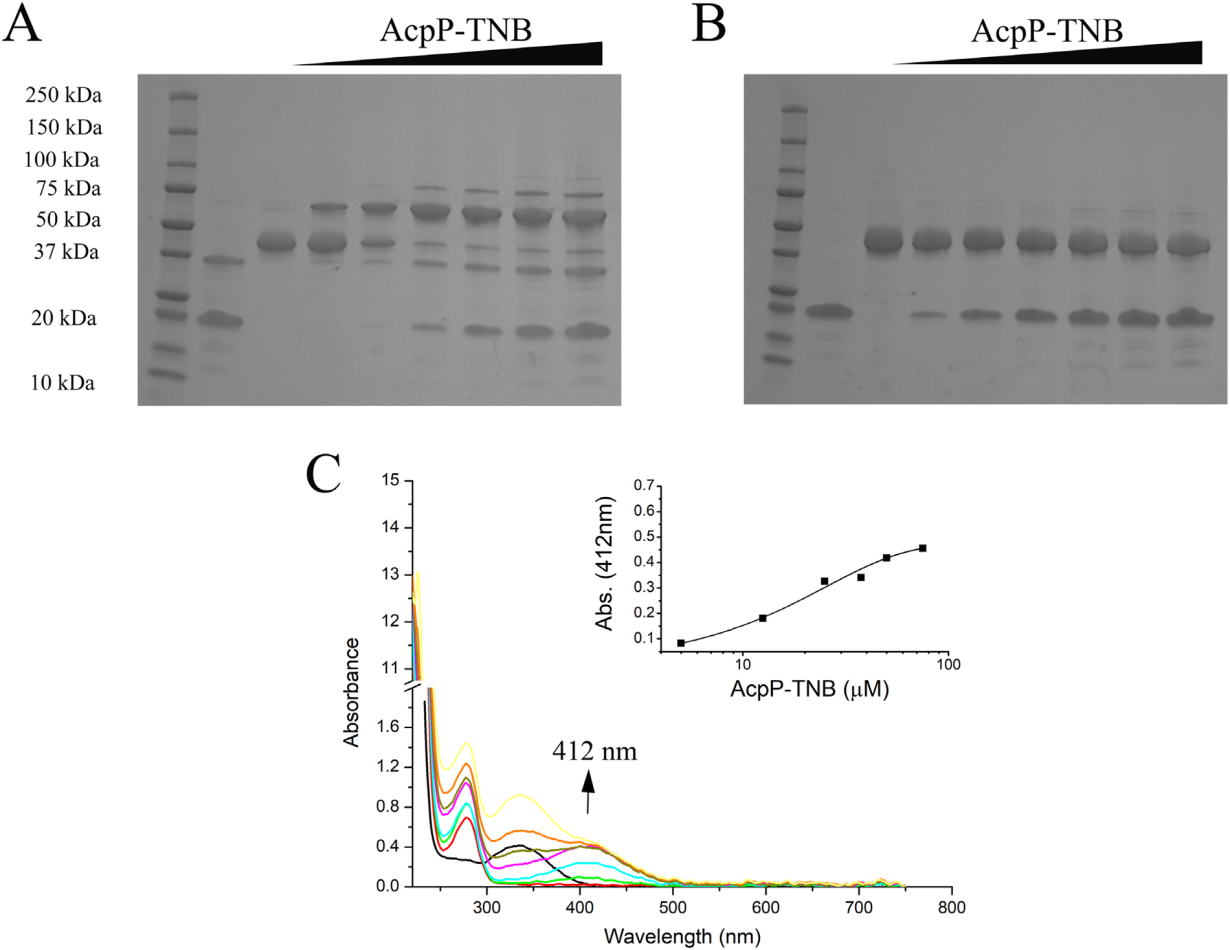
Analysis of the AcpP-TNB^−^ reaction with FabF reveals the formation of AcpP-FabF complex and concomitant release of TNB^2−^. Sodium dodecyl sulfate polyacrylamide gel electrophoresis (SDS PAGE) under non-reducing (**A**) and reducing conditions (**B**) indicate that the AcpP-FabF complex is selectively formed under non-reducing conditions. Ladder (lane 1), AcpP-TNB^−^ (lane 2), FabF (lane 3), AcpP:FabF mixed in molar ratio of 0.2:1 (lane 4), 0.5:1 (lane 5), 1:1 (lane 6), 1.5:1 (lane 7), 2:1 (lane 8) and 3:1(lane 9). The release of TNB^2−^ upon AcpP-FabF complex formation is observed by an increase in A_412_. (**C**) AcpP-TNB^−^ (black); FabF (red); 0.2:1 (green); 0.5:1 (cyan); 1:1 (magenta); 1.5:1 (dark yellow); 2:1 (orange); and 3:1 (yellow). Inset shows the increase in A_412_ versus concentration of AcpP-TNB^−^ added to FabF (average of two experiments).

**Figure 4:**
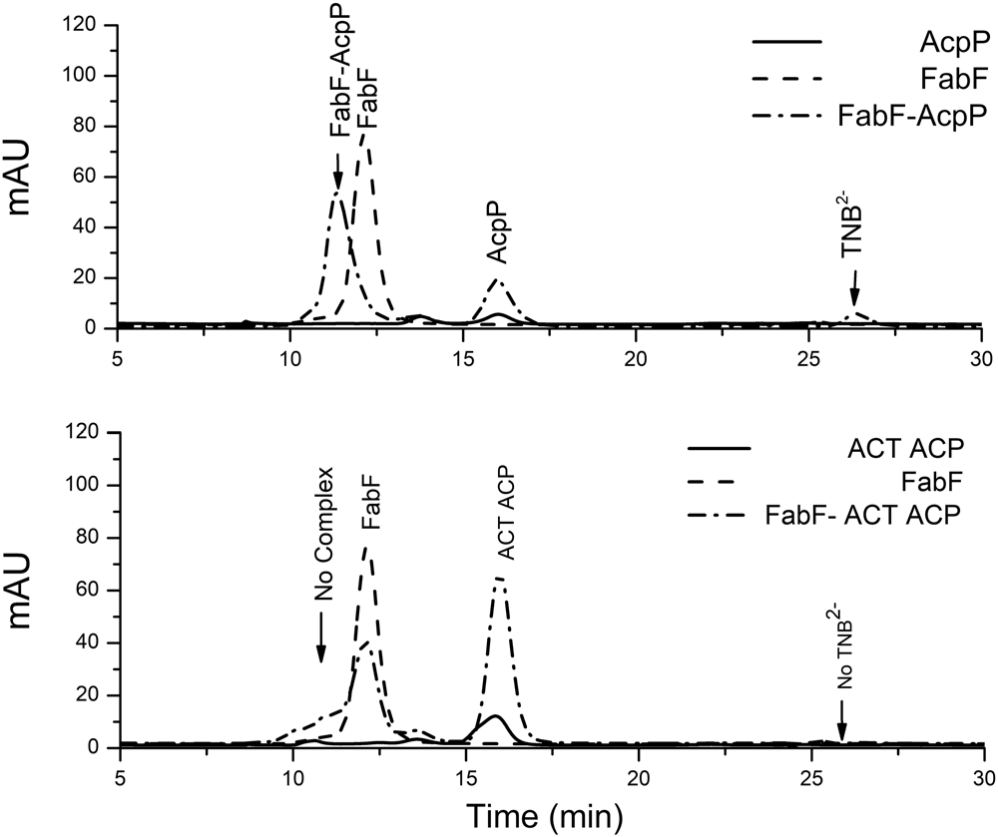
Size-exclusion chromatography (280 nm) shows that when AcpP-TNB^−^ is mixed with FabF (top), a higher molecular weight complex is formed (11 min) and the small molecule TNB^2−^ is released (26 min). Upon mixing FabF with ACT ACP-TNB^−^ (bottom), neither the higher molecular product nor TNB^2−^ is observed. ACPs alone were loaded in *holo*-form, which display a lower A_280_ than the corresponding ACP-TNB^−^ adducts.

We obtained pure AcpP-TNB^−^ and mixed up to three molar equivalents of this protein with FabF. Analysis of the potential ACP-KS cross-link via non-reducing SDS PAGE indicated indeed a larger molecular weight complex was formed of ~65 kDa (Fig. 3A), which is the same size as the previously reported AcpP-FabF complex^6^. The mixing of FabF with increasing amounts (0–3 molar equivalents) of AcpP-TNB^−^ resulted in a decrease in the intensity of the bands corresponding to the standalone proteins and increase in the putative cross-linked heterodimer. Addition of the reducing agent betamercaptoethanol (BME) led to the dissociation of this complex into two bands of ~20 and 45 kDa (Fig. 3B), consistent with the higher molecular weight complex being linked by the redox-sensitive disulfide bond. We speculate that the faint band at ~75 kDa observed under non-reducing conditions could represent two AcpPs, the second docking onto FabF in a non-mechanistically relevant manner, although further investigation is required to confirm the identity of this band. Analysis of the reaction components and products by SEC further confirmed that the ACP-TNB^−^ and FabF combined to form a higher molecular weight complex (11 min) thus releasing a small molecule product with a λ_max_ at 412 nm (27 min; Fig. 4), which is consistent with the reaction scheme outlined in Fig. 1.

To determine if the putative cross-linked product tethers the mechanistically relevant sites of ACP and FabF, the ~65 kDa band on the non-reducing SDS PAGE gel (Fig. 3A) was further interrogated by tandem proteolysis mass spectrometry. Analysis of these data revealed a peak at *m/z* = 7256 Da consistent with the trypsin-digested AcpP fragment containing the reactive site serine bound via the Ppant arm to the trypsin-digested fragment of the FabF containing the active site cysteine (Fig. S7). We also observed a decrease in counts of the unmodified, trypsin-digested fragments of AcpP containing the serine point of Ppant arm attachment and FabF containing the active site Cys163, consistent with the covalent modification of these fragments. Considering that FabF contains three additional cysteines^8^, these results highlight the regioselectivity of the ACP-FabF cross-linking reaction for the mechanistically relevant product.

The reaction of AcpP-TNB^−^ with FabF can be monitored by UV-vis because while the AcpP-TNB^−^ adduct is colorless, the TNB^2−^ ion released upon AcpP-TNB^−^ binding to FabF absorbs at 412 nm (ε = 14150 M^−1^ cm^−1^ at 25 °C)^18–20^. Given that one mole of TNB^2−^ should be released per mole of AcpP-FabF cross-link formed, the amount of ACP-FabF produced can be readily quantified using the Beer-Lambert law. As shown in Fig. 3C, an increase in A_412_ is observed as the 0–1 molar equivalents of ACP-TNB^−^ are added to FabF, and a plateau is observed upon addition of excess ACP-TNB^−^. Notably, the plateau observed at A_412_ = 0.4 corresponds to ~28 μM of TNB^2−^ released, which is consistent with the 25 μM FabF added to solution forming a cross-link with ACP at a 1:1 molar ratio. Thus, UV-vis can be used to quantitatively monitor the progress of the reaction between ACP-TNB^−^ and FabF to form ACP-FabF.

### ACT ACP-TNB^−^ does not form a mechanistically relevant cross-link with the active site cysteine of FabF

We next investigated whether the cross-linking of the ACP-TNB^−^ adduct to KS was selective to those proteins that bind *in vitro* in their *holo* form. It has previously been shown by us^6^ and others^21^ that ACT ACP does not bind to FabF, a result that we verified in this work using SV-AUC as outlined above. Upon mixing ACT ACP-TNB^−^ with FabF, we did not observe a color change, which is consistent with a lack of cross-link formation. The inability of ACT ACP-TNB^−^ to form a cross-link with FabF is supported by the lack of a higher molecular weight complex observed under non-reducing conditions (Fig. S8), a lack of increase in A_412_ observed upon mixing ACT ACP-TNB^−^ with FabF (Fig. S8c), and SEC (Fig. 4). A faint band is observed above the FabF band in the SDS PAGE gel under non-reducing conditions, which could represent non-specific, low-level binding of the ACT ACP to FabF. Nonetheless, these data support the model that the cross-linking reaction between ACP-TNB^−^ and FabF is selective for functional interactions and can be applied to screen for other hybrid ACP-KS partnerships.

### ACP-TNB^−^ can be used to screen for small molecule inhibitors of ACP-KS interactions

**Figure 5:**
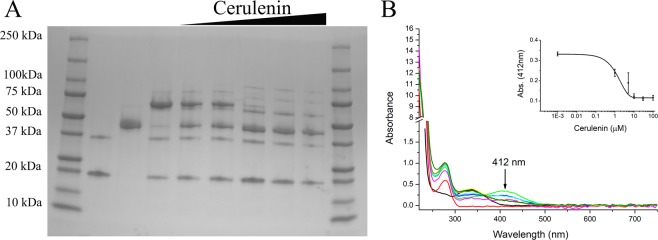
Pre-incubation of *E*. *coli* KS FabF with the active site inhibitor cerulenin (0–250 μM) prevents AcpP-TNB^−^ from forming a cross-link with FabF but also causes aggregation at higher concentrations, as observed by non-reducing sodium dodecyl sulfate polyacrylamide gel electrophoresis (SDS PAGE) (**A**) and UVvis. (**B**) Lanes: 1. Protein standards ladder; 2. AcpP-TNB^−^ (25 μM); 3. FabF (25 μM); 4–9. 25 μM AcpP-TNB^−^ mixed with 25 μM FabF preincubated with 0, 1, 10, 30, 100, and 250 μM cerulenin for 2 hours at room temperature. Inset shows the decrease in A_412_ versus cerulenin concentration (n = 3 ± standard deviation).

We reasoned that because the colored TNB^2−^ molecule is selectively released upon binding between the AcpP Ppant arm and KS active site cysteine, the methodological advance presented above could be applied to screen for small molecule inhibitors of the ACP-KS binding event, a protein-protein interaction that is essential in the construction of microbial fatty acids. To test this hypothesis, we pre-incubated FabF with cerulenin, which serves as a weak inhibitor of the active site Cys163 residue on FabF^22–24^, prior to mixing in equimolar AcpP-TNB^−^. We observed that increasing concentrations of cerulenin led to a decrease in the ACP-KS complex band on non-reducing SDS PAGE and less of an increase in A_412_ (Fig. 5). Consistent with the model that FabF can adopt multiple conformers, one of which is cerulenin-insensitive^25^, we do not observe 100% inhibition, even at concentrations in 10-fold excess. We observe a 2 μM +/− 0.5 IC_50_ value, which is consistent with the range of values reported in the literature^23,24^. These data show that the ACP-TNB^–^based colorimetric assay additionally can be used to screen for small molecule inhibitors of ACP-KS interactions.

## Discussion

Microorganisms utilize fatty acid and polyketide synthases to construct primary and secondary metabolites that serve important roles in the ability of the organism to survive and thrive. A central protein-protein interaction occurs during biosynthesis of polyketides and fatty acids between the ACP and KS^1^. These two proteins come together to facilitate two types of reactions: (1) chain transfer, which involves the active site thiolate of the KS acting as a nucleophile in a nucleophilic acyl substitution reaction of the nascent fatty acid or polyketide covalently tethered to the thiol of the ACP Ppant arm via a thioester; and (2) chain elongation, which involves a decarboxylative Claisen-like condensation of malonyl-based extender units tethered to the ACP Ppant arm via a thioester with an acyl group bound to the active site of KS via a thioester. While the ability to study mechanistically relevant ACP-KS interactions is essential to efforts to create new antibiotics through combinatorial biosynthesis and small molecule inhibitor design, progress has been stymied by the lack of methodology to easily obtain and detect ACP-KS interactions.

Incorporating a reactive electrophilic warhead, such as an epoxide or Michael acceptor, into the Ppant arm activates the ACP to form a covalent cross-link with the active site cysteine of a cognate KS in both FASs and type II PKSs^1,7–9^. Similarly, we showed previously that the modification of an ACP Ppant arm to a thiocyanate (ACP-SCN) activates the Ppant arm to form a cross-linked complex with a cognate KS, a methodology that provides easy access to the ACP-KS complex and a handle to report on functional engagement through the release of CN^−^, which can be monitored by IR spectroscopy^6^. To obtain the relevant ACP-SCN, the *holo*-ACP is first reacted with Ellman’s reagent (DNTB) to make the mixed disulfide ACP-TNB^−^, which is subsequently reacted with NaCN. In the context of an undergraduate research experience, we discovered that ACP-TNB^−^ serves to activate the ACP Ppant arm to form a complex with the thiolate active site of a cognate KS in a mechanistically relevant manner similar to what has been shown by other means by our lab and others^1,6,7,9,26^. Ppant-Cys cross-linked ACP- and peptidyl carrier protein (PCP)-protein complexes have been leveraged previously both *in vivo*^27^ and *in vitro* to obtain important information about the structure and function of ACPs and PCPs^6,28^. The discovery presented in this work is a notable advancement from previous work because of the extreme ease of preparation of the activated ACP Ppant arm (a one-pot reaction of *holo*-ACP with DNTB) as well as the ability to monitor productive engagement in real time via a visible release of TNB^2−^, which has a strong absorbance at 412 nm.

Since its introduction in 1959, DTNB (aka Ellman’s reagent) has been extensively used as an efficient and inexpensive method for the quantification of protein sulfhydryls and enzyme activity^18,29,30^. While DTNB has been applied to quantify the ratio of *apo*- to *holo*-ACPs in solution^31,32^, the application of DTNB to activate the Ppant arm of ACPs to obtain and visualize ACP interactions with proteins harboring an active site thiol represents an innovative use of this reagent. The resulting assay is an improvement from other methods to assess ACP-KS interactions because it is simpler, quicker, cheaper, and less toxic. It is also notable that this discovery emerged from a CURE experience involving undergraduate students engaged in exploring real research questions and learning from unexpected outcomes in the context of STEM education^33,34^.

The crosslinking methodology presented in this work can be expanded to investigate the interactions of other pantetheine-based moieties, such as coenzyme A and the PCPs of non-ribosomal peptide synthetases. Future efforts will focus on leveraging this methodology to obtain crystal structures of ACP- and PCP-protein complexes to identify the molecular recognition features that guide functional ACP and PCP interactions, as well as demonstrating the high throughput applications and generalizability of the colorimetric assay. There remains unmet need for a facile and high throughput method to evaluate ACP- and PCP-protein interactions from the perspectives of both the rapid screening of functional hybrid protein partnerships as well as small molecule inhibitors of FASs. We envision that the methodological advancement presented herein will help fill this gap and be useful in the assembly of synthetic pathways leading to new “unnatural natural” products as well as in the discovery and development of novel antibiotics.

## Methods

### Experimental methods

#### Cloning, expression, and purification of FabF, holo-AcpP, and holo-ACT ACP C17S

AcpP *N*-terminal His_6_-tag, AcpP *N*-and *C*-terminal His_6_-tag, and FabF (*C*-terminal His_6_-tag) were expressed from pKJ5535^35^ or pTL14^36^, and pXY-FabF^36^, respectively. UNIPROT accession numbers for the proteins used in this work are P0A6A8 (*E*. *coli* AcpP); Q02054 (ACT ACP); and P0AA16 (FabF). To express ACT ACP C17S to direct the regiospecificity of TNB^−^ addition, pKJ5573 was constructed via Q5 site-directed mutagenesis protocol (New England BioLabs) using primers okj896f, 5′ CCTCGTGGAGAGCGCCGGTGAG 3′ and okj897r 5′ GCGCGGCGCAGATCGTCG 3′ with pMC002067 (a generous gift from Professor Michelle Chang’s Lab, University of California, Berkeley) as template DNA. The C17S ACT ACP construct was used for mechanistic crosslinking and circular dichroism experiments, whereas the wild type ACT ACP was used for sedimentation velocity experiments. *E*. *coli* BAP1 cells^10^ containing pKJ5535, pTL14 (AcpP), and pKJ5573 (ACT ACP C17S) were cultured in LB media, supplemented with appropriate antibiotics, 50 μg/mL kanamycin for pKJ5535, pTL14 and 100 μg/mL carbenicillin for pKJ5573. pXY-FabF, kanamycin-resistant, was transformed into *E*. *coli* BL21 cells. Cultures (800-mL) were inoculated with overnight seed culture at volume ratio (1:200 v/v), shaken at 37 °C and 200 rpm until the OD (600 nm) 0.6–0.8 was reached. After induction with 250 μM isopropyl β-D-1-thiogalactopyranoside (IPTG), the temperature of incubation was reduced to 18 °C for an additional 12–16 hours. Cells were harvested by centrifugation at 4,500 *g* for 15 minutes, and resuspended in an ice-cold lysis buffer (50 mM sodium phosphate, pH 7.6, 300 mM NaCl, 10 mM imidazole, and 10% (v/v) glycerol) prior to lysis via sonication on ice using an XL-200 Microson sonicator (7 × 30 sec continuous pulse alternating with 30 sec rest, power level = 12 W). Cell debris were removed by centrifugation 17,000 *g* for 45 minutes. The supernatant was equilibrated with 3 mL Ni-NTA affinity resin (Gold Bio, pre-equilibrated with lysis buffer) for 2 hours at 4 °C.

Prior to applying the eluant onto a poly-prep (Bio Rad) column, 200 μL of expressed ACPs equilibrated resin was removed for analysis by LC-MS to evaluate the ratio of *apo*- vs *holo*-ACP in solution. In brief, the 200 μL sample was centrifuged at 12,470 *g* for 1 minute using a microcentrifuge to separate the supernatant, which contained lysis buffer with other proteins, from the resin-bound ACP. After washing the resin twice with 200 μL wash buffer (50 mM sodium phosphate, pH 7.6, 300 mM NaCl, and 30 mM imidazole), proteins were eluted with elution buffer (50 mM sodium phosphate, pH 7.6, 100 mM NaCl, and 300 mM imidazole). The eluted sample (10 μL) was mixed with 90 μL of ddH_2_O and used for LC-MS (see details below).

When necessary, ACPs were converted to 100% *holo* using an on-resin Sfp reaction protocol developed in our laboratory. Ni-NTA resin harboring the His_6_-tagged ACP was washed with lysis buffer until the A_280_ reached baseline and then washed again using wash buffer until the A_280_ once again reached zero. Resin was then mixed with 2 mL of an Sfp reaction mixture containing 50 mM sodium phosphate, pH 7.6, 10 mM MgCl_2_, 2.5 mM coenzyme A (CoA), 15 mM fresh dithiothreitol (DTT), and 6 μM Sfp R4–4 phosphopantetheinyl transferase (a generous gift from Jun Lin’s Lab at Georgia State University)^13^. The Sfp-catalyzed addition of the Ppant arm onto remaining *apo*-ACP took place on column upon gentle rotation at room temperature. After reacting for 12–16 hrs, the resin was washed again with washing buffer until the excess coenzyme A was removed from the reaction and A_260_ reached baseline. The Ni-bound *holo*-ACP sample was then eluted in 1-mL fractions with 10 mL of elution buffer (50 mM sodium phosphate, 100 mM NaCl, 300 mM imidazole, pH 7.6). Protein content was assessed by A_280_ readings (ε_280 nm_ AcpP = 1,490 M^−1^ cm^−1^, ε_280 nm_ ACT ACP C17S mutant = 4,470 M^−1^ cm^−1^) using a Nanodrop 2000 spectrophotometer (Thermo Fisher Scientific) and methods described in Pace *et al*. (Tyr = 1490 M^−1^ cm^−1^, Trp = 5500 M^−1^ cm^−1^)^37^. Full conversion of ACPs to their *holo* form was confirmed by LC-MS (see below). Fractions containing *holo*-ACPs were dialyzed against 50 mM sodium phosphate, pH 7.6 (3 K MWCO Slide-a-Lyzer Dialysis Cassettes; ThermoFisher), concentrated to ~1 mM using Amicon Ultra-4 3 K MWCO centrifugal filters, and stored at −80 °C for future use. FabF (KS) was purified in a similar manner as ACPs without the Sfp R4-4 phosphopantetheinyl transferase treatment. Protein content in elution fraction was assessed by A_280_ readings (ε_280 nm_ FabF (KS) = 25,900 M^−1^ cm^−1^) using a Nanodrop 2000 spectrophotometer (Thermo Fisher Scientific). Fractions containing FabF (KS) were pooled together concentrated to about 400 μM using Amicon Ultra-4 10 K MWCO centrifugal filters and stored in −80 °C with 10% (v/v) glycerol until future use. In order to prevent aggregation, stocks of FabF were dialyzed against 50 mM sodium phosphate pH 7.6 prior to each assay.

#### Addition of the TNB^−^ Moiety to holo-ACPs

*Holo*-ACPs (0.8–1.1 mM in 50 mM sodium phosphate buffer, pH 7.6) were treated with 8 molar equivalents of 25 mM 5,5′-dithiobis-(2-nitrobenzoic acid) (DTNB) in 50 mM sodium phosphate buffer, pH 7.6, for 1 h at room temperature. As shown previously, DTNB reacts with the terminal thiol of the Ppant arm of ACPs, yielding mixed disulfide ACP-TNB^−^ species and a bright yellow-colored compound, 2-nitro-5-thiobenzoate ion (TNB^2−^)^6^. ACP-TNB^−^ was isolated and desalted using a Sephadex G-25 PD-10 desalting column (GE Healthcare #17085101) pre-equilibrated with 50 mM sodium phosphate buffer, pH 7.6. A maximum of 1.5 mL DTNB reaction sample was adsorbed prior to eluting with 3.5 mL of 50 mM sodium phosphate buffer, pH 7.6. Fractions (1 mL) were collected and analyzed by monitoring A_280_ (protein) and A_340_ (ACP-TNB^−^) using a Nanodrop 2000 spectrophotometer (Thermo Fisher Scientific). Fractions containing purified ACP-TNB^−^ were pooled, analyzed by LC-MS (see below) and stored at −80 °C until further use.

#### Liquid Chromatography-Mass Spectrometry (LC-MS)

Molecular mass of *apo*-, *holo*-, and TNB^–^modified ACPs were analyzed by LC-MS using a quadruple G6125BW liquid chromatography mass spectrometry (LC-MS) instrument from Agilent equipped with a Waters XBridge Protein BEH C4 Column (300 Å, 3.5 μm, 2.1 × 50 mm) heated to 45 °C for analysis by electrospray ionization mass spectrometry (ESI MS) in the positive mode. Protein samples were eluted using the following conditions: 0–1 min 95% A, 3.1 min 5% A, 4.52 min 5% A, and 4.92–9 min 95% A (Solvent A = 99.9% water +0.1% formic acid; Solvent B = 99.9% acetonitrile +0.1% formic acid). The obtained mass spectra were deconvoluted using ESIprot,^38^ and the observed and calculated molecular weights (MWs) were compared to confirm successful phosphopantetheinylation or addition of the TNB^−^ moiety.

#### Cross-linking of ACP-TNB^-^ with FabF

25 μM AcpP-TNB^−^ or C17S ACT ACP-TNB^−^ was mixed with FabF in 50 mM sodium phosphate buffer (pH 7.6) in the following ACP-TNB:FabF molar ratios: 0.2:1, 0.5:1, 1:1, 1.5:1, 2:1 and 3:1. The reaction mixtures (20 μL) were incubated for 20 min at room temperature without shaking. Cross-linking was analyzed by SDS- PAGE, 4–20% gradient gels, Mini-PROTEAN TGX Tris-Glycine from BioRad. For non-reducing conditions, 2X loading dye (100 mM Tris-HCl, pH 6.8, 4% (w/v) SDS, 0.2% (w/v) bromophenol blue, 20% (v/v) glycerol) was used. For reducing samples, 2X loading dye was mixed with freshly prepared 5% beta-mercaptoethanol. SDS-PAGE gels were run at 120 V for ~65 min using 1X SDS running buffer (10X buffer recipe: 1% SDS, 1.92 M glycine, 0.25 M Tris base, pH 8.3). A_412_ of the reaction mixture was acquired using NanoDrop 2000 spectrophotometer (Thermo Scientific). SDS-PAGE gels were imaged using FluoroChem SP imager from AlphaInnotech, and UV-vis spectra were plotted in Origin (v.8.6.0).

#### Fast Protein Liquid Chromatography (FPLC)

AcpP-TNB^−^, C17S ACT ACP-TNB^−^, and FabF (diluted to ~1 mg/mL for FabF and ~0.5 mg/mL for *holo*-ACPs in 500 μL in sodium phosphate buffer, pH 7.6) were subjected to gel filtration using an ÄKTA pure chromatography system (GE Healthcare Life Sciences) equipped with a Superdex 75 Increase 10/300 GL column (GE Healthcare Life Sciences) with a molecular weight range of 3000–70000 Da. Cross-linked samples were run at 1:2 FabF:ACP-TNB^−^ ratios (25:50 uM). Elution was carried out in 1 mL fractions with 1.50 column volumes (CV) of the same buffer at a flow rate of 0.8 mL/min. Chromatograms were analyzed using the associated UNICORN software package and plotted in Origin (v.8.6.0).

#### Cerulenin inhibition experiment

A 7.6 mM cerulenin (Cayman Chemicals) stock solution was prepared in 1 mL of anhydrous ethanol. A total of 30 μL sample was prepared keeping FabF and AcpP- TNB^−^ at 1:1 ratio (25 μM:25 μM) with varying cerulenin concentrations (0, 1, 10, 30, 100, 250 μM). The amount of ethanol in the sample was kept constant at 3% (v/v). FabF was incubated with cerulenin for 2 hours at room temperature with gentle shaking. For high cerulenin concentration (100 and 250 μM), protein aggregation was observed. After 2 hours, AcpP-TNB^−^ was added to the 25 μM FabF and samples were incubated at room temperature for an additional 20 minutes prior to measuring the absorbance. Samples were mixed with equal volume of 2X sample buffer and loaded onto a 4–20% gradient gel. Gels were run at 120 V for about an hour until the front dye reached the bottom. Gels were washed with ddH_2_O for 5 minutes then stained with GelCode Blue Safe Protein Stain (ThermoFisher Scientific) for 15 minutes. Gel was washed overnight with ddH_2_O prior to being imaged. Absorbance data were plotted in Origin (v.8.6.0).

#### Tandem Proteolysis Mass Spectrometry

Liquid chromatography tandem mass spectrometry (LC-MS/MS) analysis was performed by the Proteomics and Metabolomics Facility at the Wistar Institute using a Q Exactive Plus mass spectrometer (ThermoFisher Scientific) coupled with a Nano-ACQUITY UPLC system (Waters). Samples from non-reducing SDS PAGE gels were digested in-gel with trypsin and injected onto a UPLC Symmetry trap column (180 μm i.d. × 2 cm packed with 5 μm C18 resin; Waters). Tryptic peptides were separated by reversed phase high pressure liquid chromatography on a BEH C18 nanocapillary analytical column (75 μm i.d. × 25 cm, 1.7 μm particle size; Waters) using a 95 min gradient formed by solvent A (0.1% formic acid in water) and solvent B (0.1% formic acid in acetonitrile). Blank gradients (30 minutes each) were run between sample injections to minimize carryover. Eluted peptides were analyzed by the mass spectrometer set to scan *m/z* from 400 to 2000 in positive ion mode. The full MS scan was collected at 70,000 resolution followed by data-dependent MS/MS scans at 17,5000 resolution on the 20 most abundant ions exceeding a minimum threshold of 20,000. Peptide match was set as preferred; exclude isotopes option and charge-state screening were enabled to reject singly and unassigned charged ions. Peptide sequences were identified using MaxQuant 1.5.2.8. MS/MS spectra were searched against a custom *E*. *coli* UniProt protein database containing the *E*. *coli* FabF and AcpP protein sequences using full tryptic specificity with up to two missed cleavages, static carboxamidomethylation (57.02146) of Cys, variable oxidation (15.99491) of Met, and variable mass addition of 339.07797 or 364.07321 on Ser, and variable presence of *N*-term Met. Consensus identification lists were generated with false discovery rates of 1% at protein, peptide and site levels.

#### Far UV-Circular Dichroism (CD)

CD spectra were collected using an Aviv Circular Dichroism Spectropolarimeter (Model 410). Spectra were collected using a High Precision Quartz SUPRASIL cuvette with 0.1- cm pathlength (Hellma Analytics) at 25 °C using a bandwidth 1 nm, 0.5 nm step size, and 3 seconds averaging time. Prior to the acquisition of spectra, large non-specific aggregates were removed using a 0.2 μm low protein-binding filter with HT Tuffryn membrane (Pall Corporation). For data analysis, protein ellipticity, mdeg, was converted to molar ellipticity, [θ], with units degrees cm^2^ dmol^−1^. The spectra were smoothed using a smoothing function implemented in the Aviv software, applying a window width of 11 data points, degree 2. Data were plotted in Origin (v. 8.6.0).

#### Analytical Ultracentrifugation, Sedimentation Velocity experiment (SV-AUC)

All experiments were performed using a Beckman model Optima XL-A Analytical Ultracentrifuge (AUC) equipped with an An-60 Ti rotor. Sedimentation velocity runs used two-channel Epon, charcoal-filled centerpieces with 1.2 cm path length containing 400 μL sample and 410 μL dialysis buffer (20 mM Tris-HCl, pH 8.0, 150 mM NaCl, and 1 mM DTT) as a reference. Sedimentation boundaries were measured at a speed of 42,000 rpm for FabF alone and in complex with ACPs, and 50,000 rpm for ACPs alone. All measurements were performed at 20 °C using a step size of 0.003 cm, a time delay of 0 s, and a total of 70–100 scans. Samples were monitored at 280 nm with a required starting absorbance between 0.3 and 1.0.

Temperature-corrected partial specific volumes (as weight-averages), densities, and viscosities were calculated using Sednterp (beta version)^39^. Heterogeneity of the mixtures was determined by using the dc/dt method^40^ implemented in DCDT+ (v.2.4.0), and a model-independent, continuous c(s) distribution using the 1 discrete component model in Sedfit (v.15.3)^41^. The 1 discrete component model takes into consideration small molar mass components that do not sediment even at 50,000 rpm^41^. Confidence intervals for temperature-corrected sedimentation coefficient, s(20,w), diffusion coefficient, D(20,w), and molecular mass (kDa) were computed using a bootstrapping method, confidence probability level 90% (±1.65 sigma) in DCDT+ (v.2.4.0)^40^. Data, fits, and residual distributions were plotted using the Gussi interface (v. 1.3.2)^42^ implemented in Sedfit (v.15.3).

A concentration-dependence profile was first established for each protein alone (FabF at 10–36 μM, *holo*-AcpP at 345–488 μM, and *holo*-ACT ACP (wild type) at 98–196 μM, all in 20 mM Tris-HCl, pH 8.0, 150 mM NaCl, and 1 mM DTT). For titration experiments, the concentration of FabF was kept constant (16 μM) and ACP concentrations varied (AcpP at 0–230 µM and ACT ACP_t_ at 0–250 μM). Sedimentation coefficient, c(s), distributions were generated using Sedfit (v.15.3) and plotted using Gussi (v.1.3.2). The effective particle theory (EPT) fast isotherm was constructed by defining the reaction boundary to be between 4 and 8 S, and then by integration of the c(s) distribution between 1 and 8 S. EPT s(w) fast and s(w) isotherms were globally fit to an A + B ↔ AB hetero-association model implemented in Sedphat (v.14.0)^43^.

## Supplementary information

Supplementary Information

## Data Availability

The data underlying the findings of this study are available from the authors upon request.
