# Supplementary material for: Colorimetric Assay Reports on Acyl Carrier Protein Interactions
Source: Sci Rep. 2019 Oct 30;9:15589. doi: 10.1038/s41598-019-51554-6 (PMC6821831; doi:10.1038/s41598-019-51554-6)
Supplement: Supplementary file 1 — Supplementary Information [file 41598_2019_51554_MOESM1_ESM.pdf]

Supporting Information

Colorimetric Assay Reports on Acyl Carrier Protein Interactions

Kofi K. Acheampong,[1] Bashkim Kokona[1] Gabriel A. Braun,[1] Danielle R. Jacobsen,[2] Karl A. Johnson,[2,*] Louise K. Charkoudian[1,*]

[1]Department of Chemistry, Haverford College, Haverford, PA 19041-1391, USA
[2]Department of Biology, Haverford College, Haverford, PA 19041-1391, USA

*Correspondence and requests for materials should be addressed to K.A.J. (kjohnson@haverford.edu) and L.K.C. (lcharkou@haverford.edu)

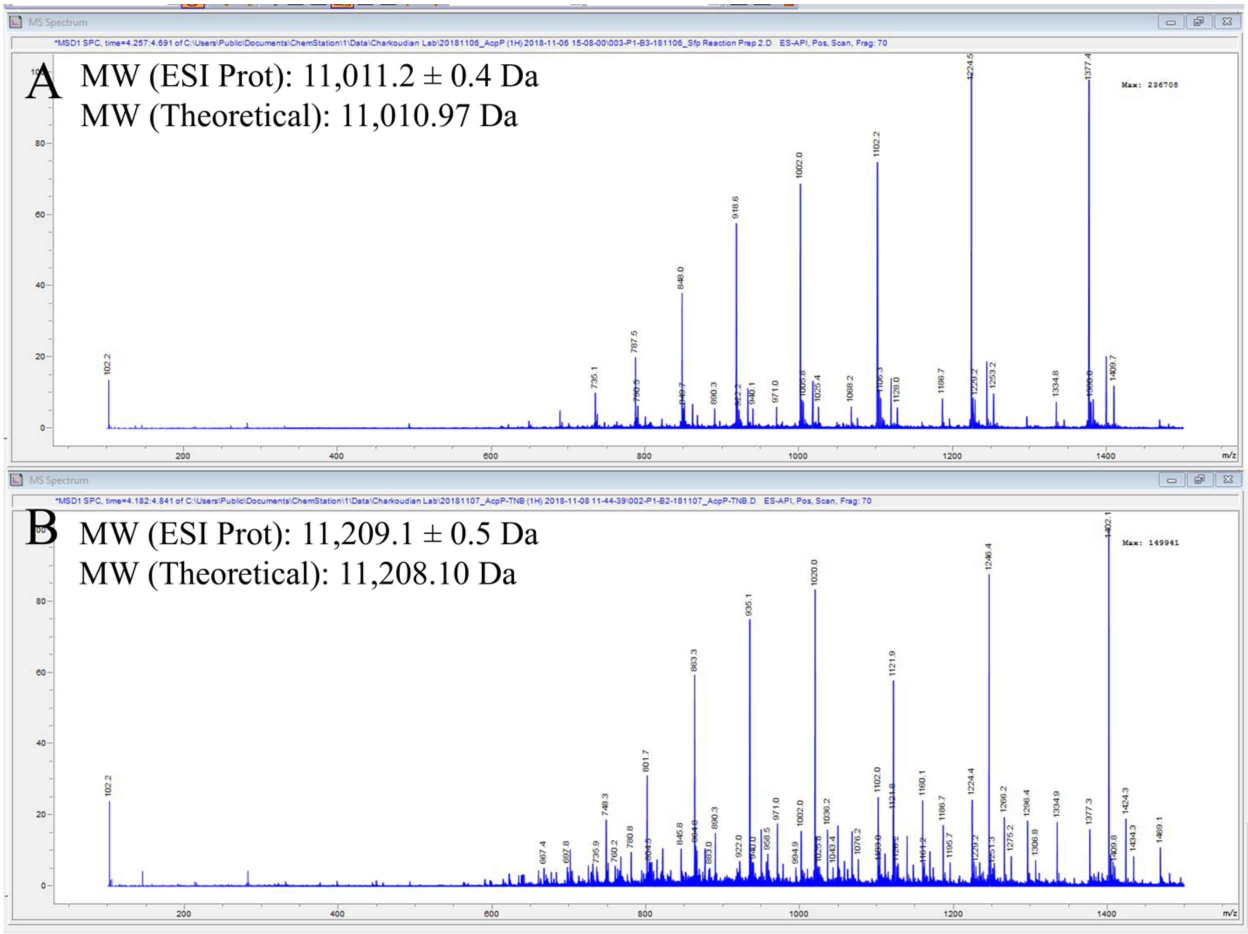

Figure S1. ES mass spectrum of *holo*-AcpP (A) and AcpP-TNB$^-$ (B). The different charge states (z) are marked on the plot and are calculated according to the theoretical molecular weight of the ACP (minus the *N*-terminus methionine). The addition of + 197.9 Da is consistent with the loading of TNB$^-$ onto the ACP Ppant arm.

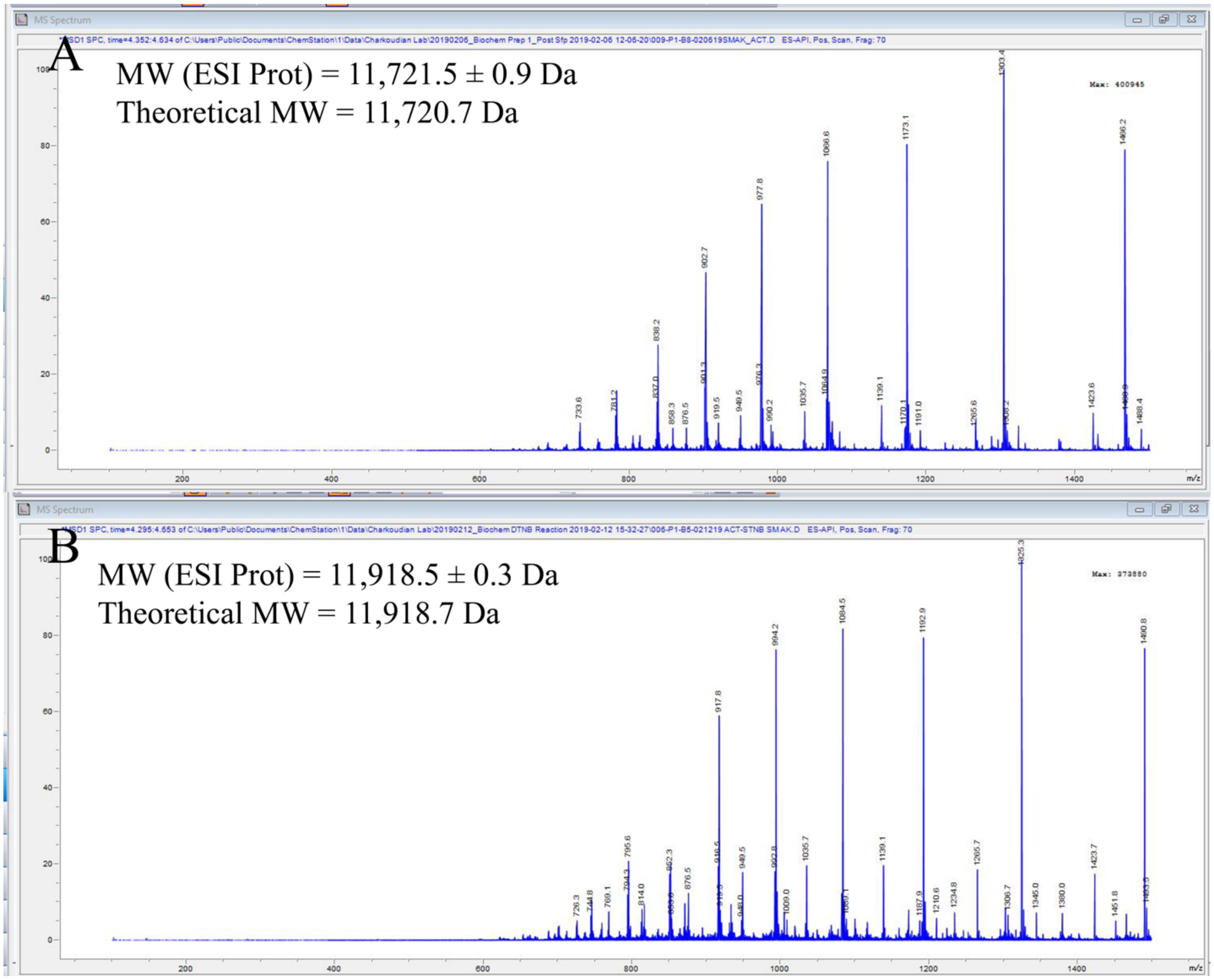

Figure S2. ES mass spectrum of *holo*- C17S ACT ACP (A) and ACT ACP-TNB$^-$ (B). The different charge states (z) are marked on the plot and are calculated according to the theoretical molecular weight of the ACP (minus the *N*-terminus methionine). The addition of + 197.9 Da is consistent with the loading of TNB$^-$ onto the ACP Ppant arm.

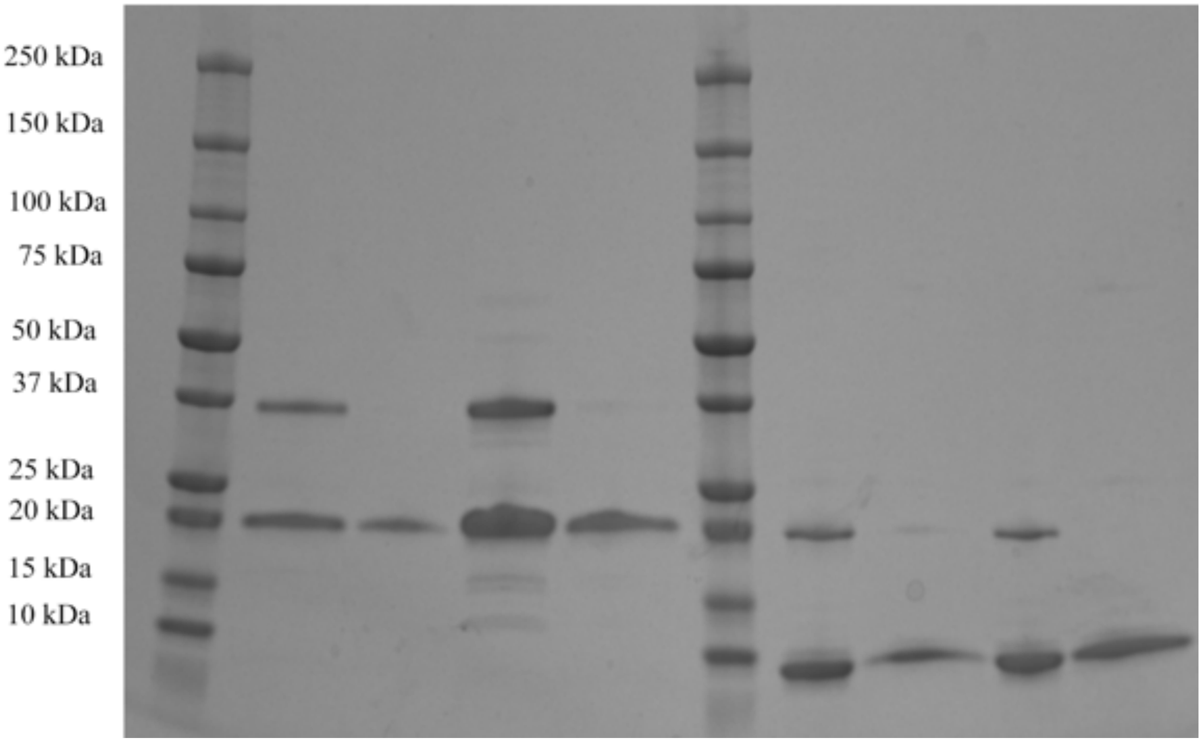

Figure S3. Sodium dodecyl sulfate polyacrylamide gel electrophoresis of acyl carrier proteins used in this study. Lanes: 1. Protein standards ladder; 2 h*olo*-AcpP (-BME); 3. *holo*-AcpP (+BME); 4. AcpP-TNB$^-$ (-BME); 5. AcpP-TNB$^-$ (+BME); 6. Protein standards ladder; 7. *holo*-ACT ACP (-BME); 8. *holo*-ACT ACP (+BME); 9. C17S ACT ACP-TNB$^-$ (-BME); 10. ACT ACP-TNB$^-$ (+BME). The migration of *holo*-AcpP to ~ 20 kDa despite its molecular weight of ~ 10 kDa is consistent with previous observations[16] and is likely due to the unusual charge distribution of the protein. A second higher molecular weight band at ~ 40 kDa represents a dimer.[17] *Holo*-ACT ACP migrated to the expect molecular weight of ~ 10 kDa also exhibited dimer formation under non-reducing conditions.

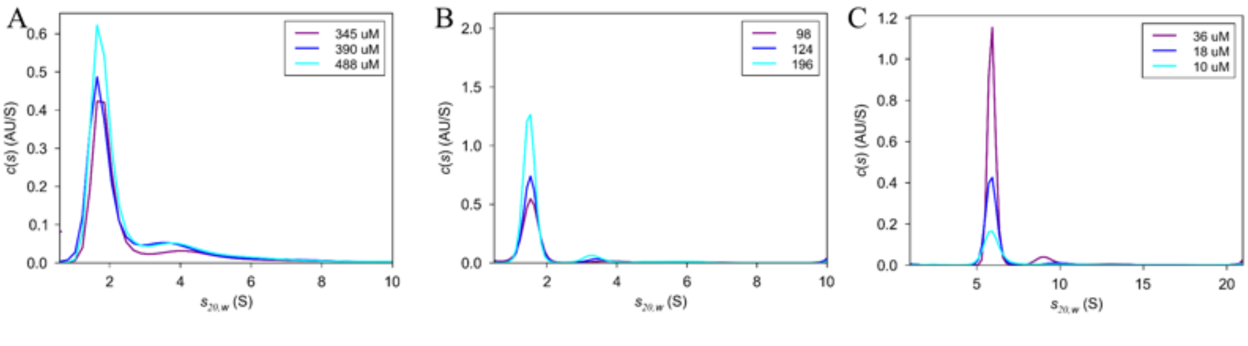

Figure S4. Oligomeric state of proteins used in this study. Sedimentation coefficient distributions c(s) of *holo*-AcpP (A) and *holo*-ACT ACP (B) shows a single peak at 1.7S and 1.5 S, respectively, which suggests that these proteins sediment as a monomer. The *E. coli* KS FabF sediments as a dimer in solution with an s-value centered at 5.9 S. There is no additional self-association observed with the increase of the monomer concentrations which suggests that all proteins used in this study exist in a single oligomeric state in solution under reducing conditions.

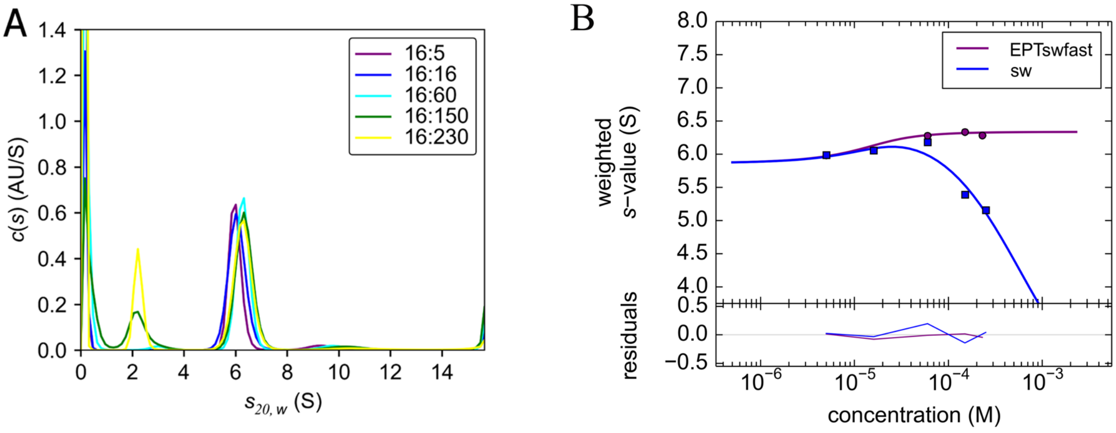

Figure S5. Binding constant for *holo*-AcpP (5-230 μM) interacting with FabF (16 μM) was determined from the concentration-dependent sedimentation velocity experiments. EPTswfast and sw isotherms of the weight-average sedimentation coefficient versus *holo*-AcpP concentration were best fitted to a A + B ↔ AB model with a binding constant k_D = 6.4 ± 1.8 μM. The AcpP peak is centered at 2.0 S in the presence of FabF. A) Unnormalized sedimentation coefficient distribution, c(s), of FabF titrated with *holo*-AcpP. B) Effective Particle Theory (EPT) weight-average sedimentation coefficient, EPTswfast (purple circles) and weight-average sedimentation coefficient isotherm sw (blue squares) plotted against *E. coli* ACP concentrations fitted to A + B ↔ AB model. The fitted isotherms are shown as solid lines. Bottom panel represent the residuals.

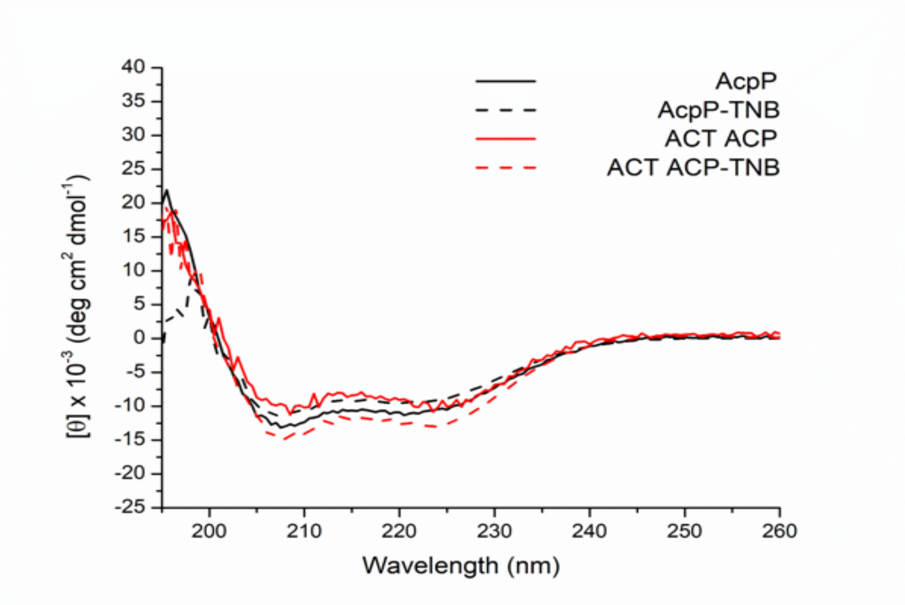

Figure S6. Far-UV CD data of *holo*-AcpP versus AcpP-TNB$^-$ (black) and *holo-* C17S ACT ACP versus C17S ACT ACP-TNB$^-$ (red) in phosphate buffer. The spectrum of ACPs in their *holo*-versus TNB$^-$-labeled form are nearly identical within the signal:noise of the instrument.

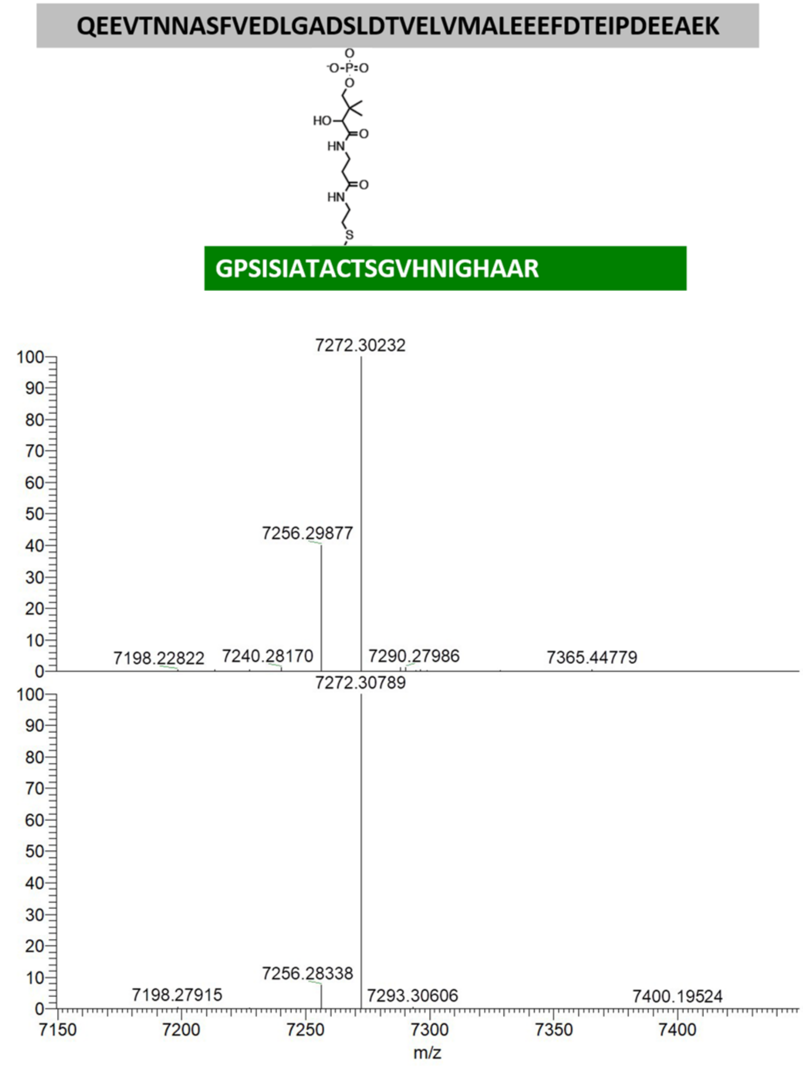

Figure S7. Zoomed in mass spectrum of the trypsinized sample from the excised ~75 kDa band from the SDS PAGE gel shown in Figure 3 representing a putative AcpP-FabF cross-link. Under dilute conditions, the excised band is appeared as a doublet and so both the top band (top panel) and bottom band (bottom panel) were analyzed by tandem proteolysis mass spectrometry. The *m/z* peak at 7256 represents the expected mass of the peptide fragment expected from a trypsinized AcpP-FabF complex linked via a disulfide bond between the thiol of the AcpP Ppant arm and the catalytic active site cysteine of FabF. The *m/z* peak at 7272 likely corresponds to the oxidized products at +16 Da.

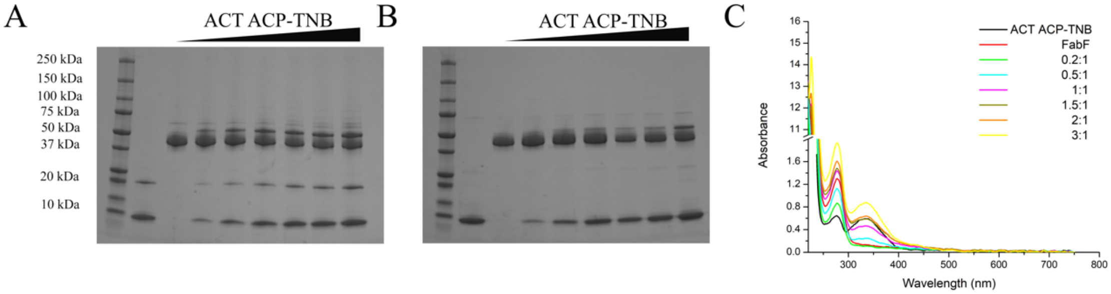

Figure S8. Analysis of the ACT ACP-TNB$^-$ reaction with FabF reveals that neither the ACT ACP-FabF complex nor the release of TNB^{2-} is observed. Sodium dodecyl sulfate polyacrylamide gel electrophoresis under non-reducing (A) and reducing conditions (B) indicate that the ACT ACP-FabF complex not formed under non-reducing conditions. Lanes: 1. Protein standards ladder; 2. ACT ACP-TNB$^-$; 3. FabF; 4. ACT ACP:FabF mixed in molar ratio of 0.2:1; 5. ACT ACP:FabF mixed in molar ratio of 0.5:1; 6. ACT ACP:FabF mixed in molar ratio of 1:1; 7. ACT ACP:FabF mixed in molar ratio of 1.5:1; 8. ACT ACP:FabF mixed in molar ratio of 2:1; and ACT ACP:FabF mixed in molar ratio of 3:1. Upon mixing ACT ACP-TNB$^-$ with FabF no release of TNB^{2-} is observed as shown by a lack of increase in A_{412} (C).
